# A predicted NEDD8 conjugating enzyme gene identified as a *Capsicum* candidate *Rf* gene using bulk segregant RNA sequencing

**DOI:** 10.1038/s41438-020-00425-7

**Published:** 2020-12-01

**Authors:** Bingqiang Wei, Paul W. Bosland, Zhenghai Zhang, Yongfu Wang, Gaoyuan Zhang, Lanlan Wang, Jihua Yu

**Affiliations:** 1 College of Horticulture, Gansu Agricultural University, 1 Yingmeng Village, Anning District, 730070 Lanzhou, China; 2 Plant and Environmental Sciences Department, New Mexico State University, P.O. Box 30003, Las Cruces, 88001 NM USA; 3 Key Laboratory of Vegetable Genetics and Physiology of Ministry of the Agriculture, Institute of Vegetables and Flowers, Chinese Academy of Agricultural Sciences, 12 Zhongguancun Nandajie, 100081 Beijing, China; 4 Vegetable Institute, Gansu Academy of Agricultural Sciences, 1 Nongkeyuan New Village, 730070 Lanzhou, China

**Keywords:** Plant breeding, Plant sciences

## Abstract

Cytoplasmic male sterility (CMS) is an important tool for producing F_1_ hybrids, which can exhibit heterosis. The companion system, restorer-of-fertility (*Rf*), is poorly understood at the molecular level and would be valuable in producing restorer lines for hybrid seed production. The identity of the *Rf* gene in *Capsicum* (pepper) is currently unclear. In this study, using bulked segregant RNA sequencing (BSR-seq), a strong candidate *Rf* gene, Capana06g002866, which is annotated as a NEDD8 conjugating enzyme E2, was identified. Capana06g002866 has an ORF of 555 bp in length encoding 184 amino acids; it can be cloned from F_1_ plants from the hybridization of the CMS line 8A and restorer line R1 but is not found in CMS line 8A. With qRT-PCR validation, Capana06g002866 was found to be upregulated in restorer accessions compared to sterile accessions. The relative expression in flower buds increased with the developmental stage in F_1_ plants, while the expression was very low in all flower bud stages of the CMS lines. These results provide new insights into the *Rf* gene in pepper and will be useful for other crops utilizing the CMS system.

## Introduction

*Capsicum* species serve as popular vegetables and spices around the world^1^. *Capsicum annuum* is the most widely grown among the five domesticated species (*C. annuum*, *C. baccatum*, *C. chinense*, *C. frutescens*, and *C. pubescens*)^1^. As with other crops, the use of F_1_ hybrid seed can greatly improve yield, resistance, and quality in peppers. The production of pepper hybrid seed requires manual emasculation, which increases the cost of seed production, and the purity of hybrid seeds produced is sometimes low.

The use of cytoplasmic male sterility and the corresponding fertility restorer is the most valuable system for exploiting hybrid vigor; it saves labor and time because it does not require manual emasculation and ensures the purity of hybrid seeds^2,3^. It has been proven that CMS is maternally inherited and is determined by mitochondrial genes resulting from chimeric ORF rearrangements that disturb the normal development of pollen^4^. However, *Rf* genes in the nucleus can override the expression of sterility or prevent the accumulation of CMS-specific gene products and in turn reverse the CMS phenotype^5–10^. It has also been suggested that *Rf* genes may be involved in the detoxification of acetaldehyde produced by ethanolic fermentation during pollen development^11^. At present, *Rf* genes have been cloned in a few species, such as rice (*Oryza sativa*)^12,13^, radish (*Raphanus sativus*)^14–18^, sorghum (*Sorghum bicolor*)^19^, and petunia (*Petunia* × *atkinsiana*)^6^. Among these *Rf* genes, most encode pentatricopeptide repeat (PPR) proteins^8,20,21^. Other types of *Rf* genes have also been cloned in rice and maize (*Zea mays*). For example, the *Rf2* genes in maize and rice encode an aldehyde dehydrogenase protein and a glycine-rich protein, respectively^11,22^, and the *Rf17* gene in rice encodes a protein of unknown function^22^. Thus, different types of *Rf* genes exist in plants.

In pepper, two CMS genes, *atp6* and *orf456*, have been studied^23,24^. It has also been reported that *orf507* from the alternate stop codon of *orf456* can inhibit the formation of microspores and result in CMS in pepper^25^. Furthermore, two CMS-specific sequence-characterized amplified region (SCAR) markers, coxII and atp6, have been developed from the sequences flanking *orf456* and *atp6-2*, respectively^26^. In addition, another molecular marker of S-cytoplasm, SCAR_130_, was reported to be more reliable than the previous markers^27^.

However, no *Rf* gene has been reported in pepper thus far. Some studies have reported that fertility restoration is regulated by one major gene. However, several cases also indicate that more complex mechanisms for fertility restoration may exist^28–30^. Additionally, partial fertility restoration of male sterility with multiple haplotypes also exists^31–33^.

As a highly conserved 76-amino-acid polypeptide, ubiquitin can be conjugated to protein substrates through ubiquitination^34^. At the initial step of ubiquitination, ubiquitin is activated by a Ub-activating enzyme (E1) through the formation of a covalent thioester bond between a cysteine residue in the E1 active site and the C-terminal end of the ubiquitin, which requires the participation of ATP. The activated ubiquitin is then transferred from E1 to a Ub-conjugating enzyme (E2) to form the E2-ubiquitin complex. Then, both the E2-bound ubiquitin and the protein substrate are connected to each terminal of a Ub ligase (E3) to form a substrate-E3-E2-ubiquitin complex. Subsequently, with the catalytic action of E3, ubiquitin is transferred to the protein substrate and forms a monoubiquitinated substrate, accompanied by the release of E2 and E3. A monoubiquitinated substrate can also have additional ubiquitin molecules added to it one by one via Lys48 of the ubiquitin molecule to form a polyubiquitinated substrate^34,35^. The ubiquitin-proteasome system (UPS) is the main degradation pathway of proteins in eukaryotic cells and plays a vital role in many biological processes^36,37^.

In addition to ubiquitin, there are some ubiquitin-like proteins (UBLs), including small ubiquitin-like modifiers (SUMOs), that act similarly to ubiquitin^38^. Among these UBLs and SUMOs, NEDD8 is the most similar to ubiquitin at both the sequence and secondary structure levels, with approximately 60% amino acid similarity identity^38–41^. Analogous to ubiquitination, the process of NEDD8 conjugation to a substrate is called neddylation and is catalyzed in a three-enzyme cascade (E1, E2, and E3)^42,43^. Neddylation plays fundamental roles in signal transduction, cell division, morphogenesis, and embryogenesis^43–46^.

By sequencing transcripts directly, the RNA-Seq method has considerable advantages for evaluating allele-specific expression, detecting novel transcripts and providing other genome-wide information from high-throughput sequencing technologies^47^. Separately, bulked segregate analysis (BSA) was developed as an efficient way to rapidly develop molecular markers for specific traits or target gene loci^48^. BSR-seq, a new method combining BSA and RNA-seq, is used not only to map traits on chromosomes but also to provide gene expression information, including information for the genes within the mapping regions^49–51^. BSR-Seq identifies differentially expressed genes (DEGs) between the two extreme-phenotype pools within the mapping interval, and the function of the candidate DEGs can be annotated by bioinformatics analysis^52^. Using the BSR-seq method, many candidate DEGs have been identified in several plant species, such as wheat (*Triticum aestivum*)^53^, cabbage (*Brassica oleracea*)^54^, and onion (*Allium cepa*)^55^.

In this study, two extreme-phenotype gene pools, a sterile pool, and a restorer pool, were constructed from F_2_ individuals of a cross between the sterile line and fertility restorer line. First, through the BSR-seq method, an approximately 16.8 M mapping region was selected, and five genes were detected to be commonly upregulated in the restorer pool and restorer line. Subsequently, only one gene, Capana06g002866, annotated as a NEDD8 conjugating enzyme E2, was cloned and chosen as a strong candidate *Rf* gene. The results provide new insights into the molecular cloning of a candidate *Rf* gene for CMS in pepper.

## Results

### SNP calling and filtering

Through SNP calling, more than 165,500 SNPs were detected in 8A, R1, SP, and RP (Table 1). Notably, the number of SNPs was higher in the restorer accessions (R1 and RP) than in sterile accessions (8A and SP) regardless of subcategory. In addition, the number of homozygous SNPs far outweighs that of heterozygous SNPs in all samples.

**Table 1:** The statistics of SNPs tested on the four accessions

Samples	Homozygous SNP	Heterozygous SNP	All SNP
8A	158822	6685	165507
R1	177864	17054	194918
SP	109666	67928	177594
RP	116680	74002	190682

**Fig. 1:**
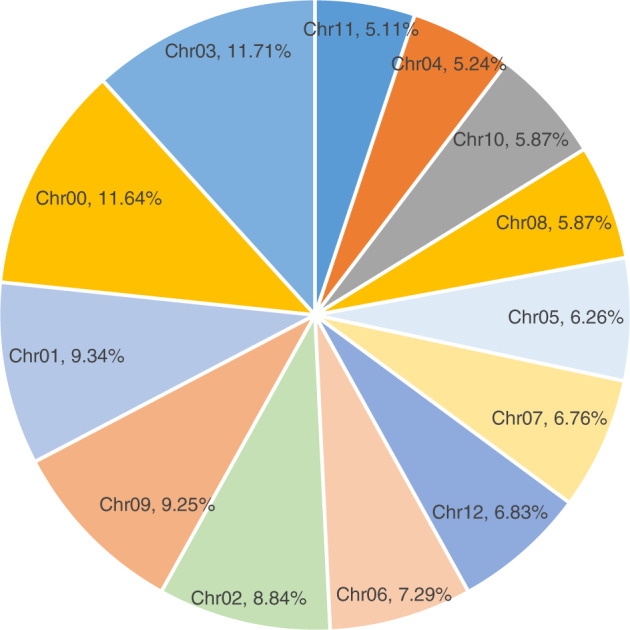
The distribution of filtered SNPs across chromosomes. Each part of the pie chart represents a chromosome and the percentage of SNPs

To ensure the accuracy of subsequent analysis, the SNPs were filtered. First, degenerate SNPs were filtered, and then the sites supported with fewer than three reads were removed. Finally, the sites where SNPs differed between the extreme-phenotype pools and its corresponding parent were filtered out. After that, the high quality and credible SNPs were retained. On the basis of these reliable SNPs, a total of 62,629 polymorphic sites between the two extreme-phenotype pools were identified, which were then used to perform the association analysis (Table 2). The SNPs were distributed on all 12 chromosomes, and some were not assigned to any chromosome (chromosome00) (Fig. 1). The chromosomes with the most SNPs were chromosome03 and chromosome00, 11.71% and 11.64%, respectively. The chromosomes with the fewest SNPs were chromosome11 and chromosome04, with 5.11% and 5.24%, respectively.

**Table 2:** The filtered SNPs from the two extreme-phenotype pools

Sample	Homozygous SNPs	Heterozygous SNPs	All SNPs
SP	6603	56026	62629
RP	5414	57215	62629

### Preliminary mapping of the *Rf* gene

**Fig. 2:**
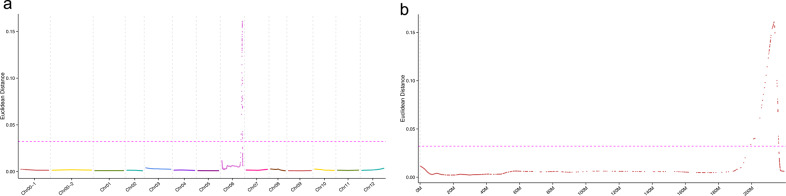
The fitting association analysis and peaks shown on chromosomes; the pink horizontal dashed line represents the significance threshold. **a** The association peaks shown on all the chromosomes. **b** Magnification of the peak showing only chromosome 6

Through the ED algorithm, only one single peak was observed to exceed the threshold significantly, on the end of chromosome06 (Fig. 2). The median + 3SD of all site fitted values, which was 0.033421494897978, was taken as the correlation threshold for analysis. An interval above the threshold was screened as the mapping interval where the *Rf* gene was located. This indicated that the *Rf* gene was mapped to an interval of 16.8 Mbp (Chr06: 199389022-216191732), close to the end of chromosome06 (Fig. 2). In addition, there was more than 76% identity between the identified fertility region and KASP genotyping of KS18 and KS22 for the 72 F_2_ individuals, which indicated that the region mapping was reliable. The mapping region was further narrowed to approximately 5.1 Mb (Chr06: 210576870–215685280) according to two SNP locations derived from the KASP markers KS18 and KS22.

**Fig. 3:**
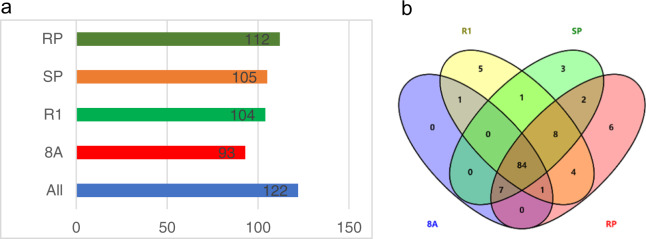
The detected genes within the mapping interval differ between four materials. **a** The number of genes detected in all and different materials. **b** Venn analysis of the genes detected in the four materials

**Fig. 4:**
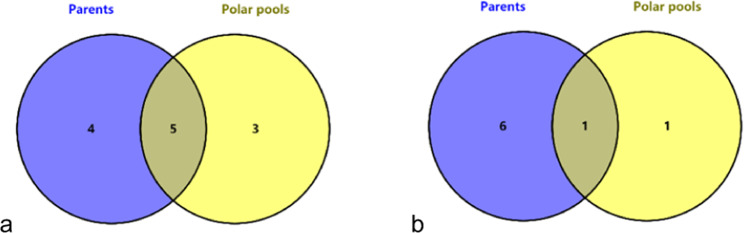
Analysis of the commonly expressed genes within the interval. **a** Venn analysis of upregulated genes in common. **b** Venn analysis of the downregulated genes in common. Parents indicate the differentially expressed genes between two parent lines, pools indicate differentially expressed genes between two extreme pools, overlap indicates the differentially expressed genes in common, and number indicates the number of differentially expressed genes

In this mapping interval, 199 genes, from Capana06g002727 to Capana06g002990 were detected, of which 93, 104, 105, and 112 genes were detected in 8A, R1, SP, and RP, respectively, and 84 genes were common to all pools (Fig. 3). Between the two extreme-phenotype pools, there were eight genes upregulated and two genes downregulated in the RP compared to the SP. To increase the range limit of candidate genes, it was necessary to identify the common upregulated and downregulated genes. Between the two parent lines, nine genes were upregulated and seven genes were downregulated in R1 compared to 8A. Interestingly, five genes (Capana06g002839, Capana06g002848, Capana06g002866, Capana06g002871, and Capana06g002913) were upregulated in both R1 and RP compared to 8A and SP, and one gene (Capana06g002754) was downregulated in both (Fig. 4, Table 3). It was also interesting that a gene (Ca06g002814) encoding a PPR protein was detected in the mapping region. The FPKM and log_2_FC values of these genes are listed in Table 3.

**Table 3:** Identification of the candidate restorer-of-fertility gene for cytoplasmic male sterility in pepper

Gene ID	8A		R1		SP		RP		log2FC	
	Counts	FPKM	Counts	FPKM	Counts	FPKM	Counts	FPKM	R1/8A	RP/SP
Capana06g002839	7.13065	0.102097	43.7989	0.627527	4.91235	0.070344	61.29.7	0.874673	2.43953	3.55695
Capana06g002848	76.2099	1.95414	167.258	4.29379	76.6083	4.65944	182.791	4.65944	1.1275	1.238
Capana06g002866	0	0	77.6972	7.43271	0	0	90.1634	8.40284	6.07699	6.16937
Capana06g002871	0	0	158.623	33.744	4.91235	1.03698	255.026	51.2701	7.10298	5.59804
Capana06g002913	61.4566	1.60394	197.788	5.16819	191.581	5.00118	360.539	9.35324	1.31663	1.02797
Capana06g002754	725.901	274.208	303.041	116.369	408.602	1078.26	95.4213	272.638	−1.251849	−1.9898
Capana06g0028144	11.2023	0.414992	0	0	12.6309	0.468067	1.32396	0.04863	−6.016956	/

### Screening and expression of the strong candidate *Rf* gene

**Fig. 5:**
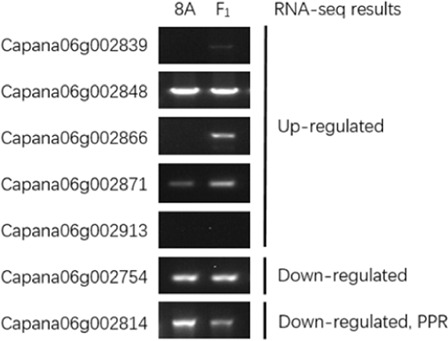
Electrophoresis of the candidate gene amplification product in 8A and F1. The expression of candidate genes differ between the flower buds of 8A and F_1_. The left collumn means the name of candidate genes, the middle electrophoresis shows the results of RT-PCR of candidate gene in the flower buds of 8A and F_1_, and the right collumn indicates the relative expressin of candidate genes in the flower buds of F_1_ compared to that of 8A by qRT-PCR method

The results of the candidate gene analysis comparing 8A and F_1_ showed that Ca06g002866 amplified the target fragments in F_1_ but not in 8A, and Ca06g002839 showed a very weak band in F_1_ but not in 8A. While four genes (Capana06g002848, Capana06g002871, Capana06g002754, and Capana06g002814) produced fragments in both 8A and F_1_, one gene, Ca06g002839, did not produce any bands in either F_1_ or 8A (Fig. 5). Therefore, we chose Ca06g002866 as the best candidate *Rf* gene in pepper. Sequencing results showed that the ORF length of Capana06g002866 is 555 bp, the same as the reference gene sequence, and encodes 184 amino acids. Functional annotation indicates that Capana06g002866 encodes a Ubc12-like NEDD8-conjugating enzyme.

**Fig. 6:**
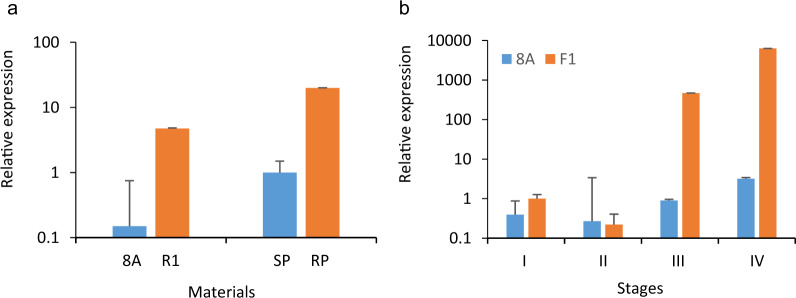
qRT-PCR analysis of Capana06g002866. **a** qRT-PCR validation in the sequenced accessions. **b** Analysis of relative expression in different developmental stages in 8A and F_1_

Subsequent qRT-PCR confirmed the differential expression of the candidate genes, and the results of qRT-PCR were identical to the sequencing results, which indicated that the expression of Capana06g002866 was obviously upregulated in restorer accessions compared to sterile accessions (Fig. 6a). Furthermore, the expression pattern indicated that the relative expression of Capana06g002866 was very low across different flower developmental stages in 8A, while it varied dramatically in the F_1_ plants (Fig. 6b). The relative expression began to increase at stage III and quickly peaked at stage IV (Fig. 6b).

## Discussion

The utilization of the CMS/*Rf* system is one of the most effective ways to produce F_1_ hybrids. The *Rf* genes encode different proteins that restore the fertility of CMS through different mechanisms, and the identity of the gene (or genes) involved in fertility restoration in pepper, as well as the mechanism through which it works, is still unclear. Clarification of the mechanism of fertility restoration for CMS would be helpful for accelerated breeding of restorer lines.

The BSR-seq method provides a powerful approach for selecting candidate genes^53–55^. In this study, BSR-seq was used to map a region of 18.6 Mbp on chromosome 06 that restores fertility. Using common upregulated DEG analysis, the five most upregulated DEGs were selected for further analysis. We examined the sequence counts and FPKM in the restorer and sterile pools. These values should be zero or very low in sterile accessions and higher in fertile accessions (Table 3). We further amplified the candidate genes within the mapping region and found that only Capana06g002866 could be cloned in F_1_ plants but not in CMS line 8A (Fig. 5), which was consistent with the requirements of the *Rf* gene. In addition, Capana06g002866 was upregulated in restorer accessions and showed very little expression in sterile accessions, which was further validated by the qRT-PCR results. It should be noted that Capana06g002866 is predicted to be a NEDD8 conjugating enzyme. Therefore, Capana06g002866 is the most likely candidate for involvement in the restoration of fertility in pepper.

The NEDD8 conjugating enzyme is the second key enzyme in the three-enzyme cascade process of neddylation^42,43^. Neddylation plays fundamental roles in signal transduction, cell division, morphogenesis, and embryogenesis^43–46^. Neddylation is also involved in pollen development, and it has been demonstrated that the NEDD8 ligase DCN1 (DEFECTIVE IN CULLIN NEDDYLATION 1) is very important in pollen development and pollen tube growth of tobacco^46^. Additionally, it has been reported that several genes related to ubiquitin ligase were upregulated in fertile anthers compared to CMS lines in pepper^56^. This indicates that neddylation is involved in pollen production and could be involved in the regulation of male fertility in pepper. Another example of ubiquitin involvement in pollen development is that the ubiquitination level of the 80 kDa protein produced in male sterile flower—in wheat decreased significantly with the development stage and reached the lowest level during the trinuclear stage^57^.

Ubiquitination function depends on the enzymatic cascade of E1, E2, and E3. The E3 ligase is a very important enzyme for conjugating the ubiquitin molecule to its substrate. HECT, RING finger, U-box, and PHD finger are the four classes of E3 ubiquitin ligases identified to date^58^. Cullin RING E3 ubiquitin ligases (CRLs) are the most prominent E3 type among the four classes^59^. Cullins are the main component of CRLs, and the activation of CRLs depends on the neddylation of cullin^37,60–62^. Notably, one of the genes upregulated in restorer lines in this study (Capana06g003066) encodes a cullin protein. With catalysis by E3, the substrates are first modified by a single ubiquitin molecule, and then other ubiquitin molecules are added one by one to form polyubiquitination^34,35^. The polyubiquitinated substrate is then recognized by the 26 S proteasome and degraded with the release of single ubiquitin molecules^34,63–65^. The ubiquitin-mediated proteasome system (UPS) relies on this well-known posttranslational modification (PTM) of proteins and is the major degradation pathway of cellular proteins in general. The UPS plays an important role in cell cycle progression, apoptosis, stress response, and growth and development processes in eukaryotes^35,66,67^. It has been reported that ubiquitination and UPS are involved in acrosome biosynthesis and sperm tail formation in human and animal spermatogenesis, UPS plays an important role in the degradation of organelles and excess protein in sperm metamorphosis, and abnormal regulation of UPS leads to sperm deformity^68–71^.

It can be inferred that the NEDD8-conjugating enzyme gene may restore the fertility of CMS through the activation of CRLs, which in turn promote ubiquitination in the restoration process. Ubiquitination is known to tag substrates for degradation by the 26 S proteasome. Neddylation facilitates ubiquitination by activating CRLs through modification on cullins, which are known to be important components of E3 for ubiquitination^72^. Because of this relationship, the increase in NEDD8-conjugating enzyme expression in R1 and RP could be interpreted as an increase that facilitates degradation. This would make sense in the context of fertility restoration if the substrate is the pollen-aborting mitochondrial ORF being tagged for degradation.

In addition, recent research clarified that neddylation is also involved in the DNA damage response and DNA repair process^37,73,74^. DNA damage is detected in the nuclei of pollen mother cells (PMCs) of CMS plants in pepper^56^. The damage may cause abnormal programmed cell death (PCD)^75^. Disturbed PCD in the tapetum will lead to a CMS phenotype^76^. These observations seem to indicate that neddylation may restore fertility by repairing DNA damage. Whether neddylation could be involved in fertility restoration through UPS or DNA repair requires further study.

**Fig. 7:**
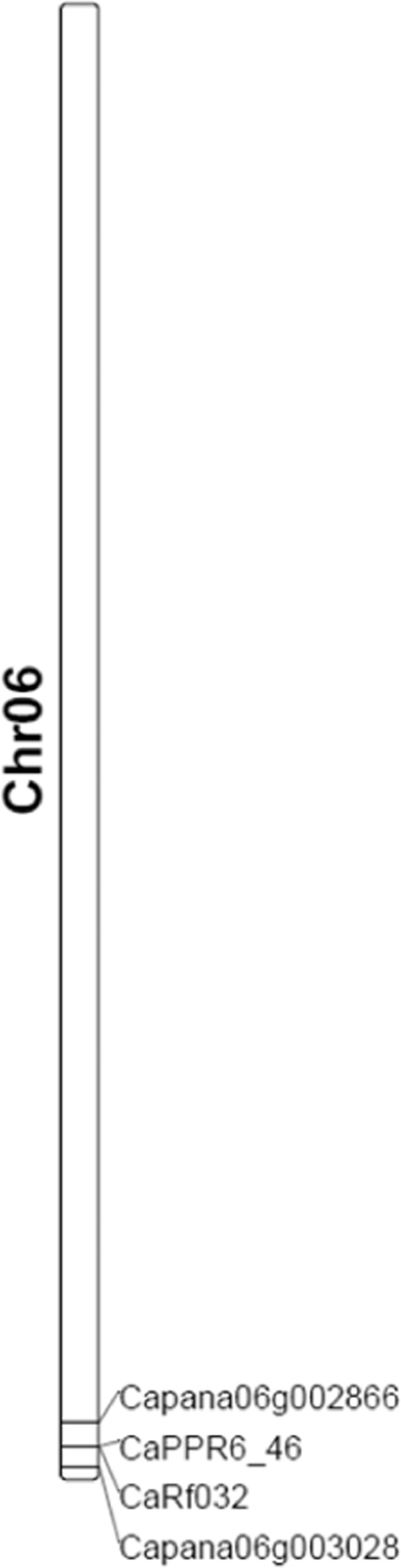
The location of several *Rf* candidate genes on chromosome 6 of the reference genome of Zunla1

It is interesting that the candidate Rf genes in our study and the previous studies were all located on the terminus of chromosome 6, such as *CaPPR_46*^77,78^, *CaRf032*^79^, and *Capa06g002866*^80^ (Fig. 7). However, the candidate *Rf* gene in our study encoded a NEDD8 conjugating enzyme, while the other candidates were mainly PPR proteins. It is worth mentioning that the majority of *Rf* genes encode PPR proteins^8,20,21^. The PPR genes are members of a protein family that is exceptionally large in plants, and pepper is no exception, with more than 550 putative PPR genes identified^77^. Some attempts have been made to find pepper *Rf* genes with a PPR structure^77,78^. In this study, it is interesting that a gene, Capana06g002814, is a predicted PPR protein that was mapped to the same region related to the restorer-of-fertility for CMS in pepper. However, Capana06g002814 was downregulated in fertility restorer accessions compared to cytoplasmic male sterile accessions and could not be detected in R1 because the sequence number and FPKM were zero. Logically, the *Rf* gene should be upregulated in restorer lines and not expressed in CMS lines. When compared to previously identified PPR genes presumed to be related to restorer-of-fertility function (CaPPR1 and CaPPR6)^73^, Capana06g002814 showed 86% and 84% similarity, respectively. Because of the nature of PPRs as a protein family are similar in structure, it is difficult to say whether this 84–86% similarity in structure indicates any similarity in function. However, this PPR protein consisted of only nine motifs, while *Rf*-like PPR proteins are generally approximately 15–20 motifs long^20^, with the CaPPR1 and CaPPR6 genes consisting of 14 PPR motifs^78^. Thus, it is more likely coincidental that this PPR gene appears in the mapping region than that it plays an actual role in fertility restoration.

## Materials and methods

### Sample, transcriptome sequencing, and DEGs

The CMS line 8A (*C. annuum* L.), the restorer line R1 (*C. annuum* L.), and two extreme pools, a sterile pool (SP) and a restorer pool (RP), were described in our previously published articles^81^. The methods of transcriptome sequencing, quantification of gene expression levels and DEG analysis were the same as in the previous articles^81^.

### SNP calling and filtering

For transcript sequencing, the reads of each sample were mapped to the reference genome using the comparison software STAR (https://github.com/alexdobin/STAR/releases), and the SNP sites were found through the SNP calling flow of GATK (https://www.broadinstitute.org/gatk/index.php) for RNA-seq. The quality value of recognition and the depth of sequencing affect the reliability of SNPs, so to obtain the most reliable SNPs, elementary screening of SNPs was carried out according to the following criteria. First, no more than 3 continuous mismatched SNPs within a 35 bp range were permitted. Second, the quality value of the SNP after the standardization for sequence depth was greater than 2.0. In addition, to identify the SNP loci differing between RP and SP for association analysis, discrepant-type SNP loci between RP and R1, as well as SP and 8A, were filtered out, and then SNP consistent-type loci between RP and SP were filtered out.

### Association analysis

The Euclidean distance (ED) algorithm calculated the region linked to the target gene^82^. In this algorithm, the depth differences of SNPs between the extreme fertile pool and extreme sterile pool calculated the ED values between two extreme pools according to the following formula:

$$\rm{ED} = \sqrt {(A_{RP} - A_{SP})^{2} + (C_{RP} - C_{SP})^{2} + (G_{RP} - G_{SP})^{2} + (T_{RP} - T_{SP})^{2}}.$$

In the formula, the higher the ED value is, the greater the difference in the SNP between the two extreme pools. The letters A, C, G, and T represent the four corresponding bases. Theoretically, the A_RP_ in the formula is the depth of base A in the extreme fertile pool, and A_SP_ is the depth of base A in the extreme sterile pool. However, in practice, the difference in sequencing quantity between mixed pools will lead to the bias of ED results. To eliminate this error, the frequency of the base at each point instead of absolute depth was used to calculate the ED value in this project. In addition, the original ED value was raised to a power of 5 (ED^5^) to decrease the background noise.

According to the linkage principle, the SNP loci nearest the real association region will tend to differ between the two extreme pools. To control the false positives caused by the association of a single locus, fitting analysis is performed depending on the location information of the SNP in the reference genomes. The fitting correlation value for each SNP was the median of the 50 up- and downstream SNPs.

### Validation of the mapping region

A KASP assay was used to further confirm the mapping region by examining the SNP flanking the restorer locus in the 72 F_2_ individuals of the cross of 8A and R1 (Table 4). Allele-specific primers for the KASP assay were designed with the online version of BatchPrimer3 V1.0 (https://probes.pw.usda.gov/batchprimer3/) and synthetized by Sangon Biotech (Shanghai) Co., Ltd. The primer pairs were composed of two forward primers, A1 and A2, and reverse primer C. The two allele-specific forward primers had the same primer sequence except for the targeted SNP at the 3′ end and their FAM and HEX-labeled tails (FAM tail: 5′-GAAGGTGACCAAGTTCATGCT-3′; HEX tail: 5′-GAAGGTCGGAGTCAACGGATT-3′), respectively. KASP assays were performed in 96-well plates with a 10 µl reaction mixture composed of 5 µl template DNA (30 ng µl^−1^), 2 µl of 2× KASP Master Mix, and 0.14 µl primer mixture according to the KASP genotyping chemistry user guide and manual (LGC Genomics, Shanghai, China). The KASP was performed on QuantStudio®5 (Applied Biosystems, US) as follows: an initial denaturation step at 94 °C for 15 min, followed by 10 touchdown cycles at 94 °C for 20 s and 61–55 °C for 60 s (dropping 0.6 °C per cycle), followed by 26 cycles at 94 °C for 20 s and at 55 °C for 60 s, and three additional cycles at 94 °C for 20 s and 57 °C for 60 s.

**Table 4:** Information on KASP primers

SNP location	Primers	
Chr06: 210576870	KS18	A1: GAAGGTGACCAAGTTCATGCTCTGGGGTGCATTCCCTTA
		A2: GAAGGTCGGAGTCAACGGATTCTGGGGTGCATTCCCTTC
		C: GTTACCTTTGTGGAATGTTGTTATGTA
Chr06: 215685280	KS22	A1: GAAGGTGACCAAGTTCATGCTCAGCGCCATCATCTTCCT
		A2: GAAGGTCGGAGTCAACGGATTCAGCGCCATCATCTTCCG
		C: GCTAAAGGCATACCAACATGGA

### Cloning of candidate genes

First-strand cDNA was obtained using a synthesis kit for first-strand cDNA (Revert Aid Premium Reverse Transcriptase) (Thermo Scientific, EP0733). Pairs of primers (Table 5) for amplification of candidate genes were designed according to the reference sequences. The PCR conditions were as follows: denaturation at 95 °C for 5 min; 35 cycles of denaturation at 95 °C for 30 s, annealing at 45–60 °C for 30 s and extension at 72 °C for 50 s–2 min; and a final extension at 72 °C for 10 min. The target PCR product was purified and recovered using the AxyPrep Gel DNA Extraction Kit (Axygen, China) and cloned into the vector pMD19-T (TaKaRa, Dalian, China) for sequencing.

**Table 5:** The primers for the amplification of candidate genes

Gene ID	Primers	Length/bp
Capana06g002839	Fw: ATGAGCAAAGAGAAGGCATTGAG Rv: TTAGTTTATCCTTCTCAAATGTGTAGACT	2379
Capana06g002848	Fw: ATGGCCAATAAAGGAGGTATACTGA Rv: TCAAGGTAACTGCTCTTGCTGAAT	1431
Capana06g002866	Fw: ATGATTAATTTGATCAAAGTAAAAGAAAA Rv: TCAATCTACAACTCGATCGAAATTC	555
Capana06g002871	Fw: ATGAATTTTGAAGTTACAATTAAACCTG Rv: TTATAGAATGCGATCGTAAGATAGGC	378
Capana06g002913	Fw: ATGGATACTGAGAAGAGACTCTATGAAG Rv: TCAAACATTGCCATAAATTGAATTT	1410
Capana06g002754	Fw: ATGTCAGGCAGAGGCAAGG Rv: CTAACCCCCAAATCCATAAAGAGT	312
Capana06g002814	Fw: ATGAAGCAGAAAGGAATTCATCC Rv: TTAGTTTTTCGAGTGAAGCTCCG	1062

### qRT-PCR analysis of Capana06g002866

The qRT-PCR primers (Capana06g002866-Fw-q: AGGGAAGATTGGAAACCTGTT; Capana06g002866-Rv-q: ATCTTTCAACTCATTGGCAGC) tested its relative expression, and *CaActin* (GenBank Accession: GQ339766.1) was chosen as an internal control (CaActin-Fw-q: TGCCTGATGGACAAGTTATTACC; CaActin-Rv-q: TGAGCACAATGTTACCGTAGAGG). qRT-PCR was carried out in a total reaction volume of 20 µL, including SG Fast qPCR Master Mix (High Rox) (2×) (BBI, B639273). qRT-PCR was performed with the following parameters: denaturation at 95 °C for 3 min, followed by 45 cycles of denaturation at 95 °C for 7 s, annealing at 57 °C for 10 s, and extension at 72 °C for 15 s on an ABI StepOne Plus Real-Time PCR System (Applied Biosystems, USA). At least three replicates were performed for all reactions.

## Data Availability

The raw sequences can be found in the Sequence Read Archive (trace.ncbi.nlm.nih.gov/Traces/sra/sra.cgi), and the temporary SRA accession number is PRJNA547768.
